# Iridocorneal endothelial syndrome in a patient with keratoconus – a case report

**DOI:** 10.1186/s12886-019-1215-x

**Published:** 2019-11-11

**Authors:** Michele De Maria, Danilo Iannetta, Antonio Moramarco, Luigi Fontana

**Affiliations:** 1 Ophthalmology Unit, AUSL-IRCCS of Reggio Emilia, Reggio Emilia, Italy; 2 Clinical and Experimental Medicine Ph.D. program, University of Modena and Reggio Emilia, Modena, Italy

**Keywords:** Keratoconus, Essential iris atrophy, Iridocorneal endothelial syndrome, Keratoplasty, Glaucoma

## Abstract

**Background:**

To describe a case of a rare association of bilateral keratoconus and unilateral essential iris atrophy and to conduct a literature review of the current strategies of treatment of the corneal disease and glaucoma in patients with Iridocorneal Endothelial Syndrome (ICE).

**Case presentation:**

We report a rare association of bilateral keratoconus and unilateral essential iris atrophy in a 38-year-old man. Diagnosis of bilateral keratoconus was confirmed by corneal topography. Slit-lamp examination showed extensive iris atrophy with corectopia and policoria in one eye. Corneal specular microscopy revealed an abnormal endothelium morphology in the same eye with extensive peripheral anterior synechiae and closure of the drainage angle at gonioscopy. Intraocular pressure was 26 mmHg, despite maximal topical therapy. Optic disc examination showed severe glaucomatous cupping. Surgery by glaucoma drainage device implantation was performed.

**Conclusion:**

Essential iris atrophy is a rare clinical variant of ICE syndrome characterized by profound anatomical alterations of the anterior segment associated with corneal edema and secondary glaucoma. In these patients, selective keratoplasties have replaced penetrating keratoplasty to treat corneal decompensation and glaucoma drainage devices are preferred to conventional trabeculectomy for the treatment of secondary glaucoma.

## Background

Keratoconus (KC) is a progressive corneal ectasia with onset typically in the second decade of life. KC progression causes visual loss as a result of increasing irregular corneal astigmatism with myopia, progressive corneal ectasia with thinning and scarring [1].

Iridocorneal Endothelial Syndrome (ICE) is a group of ophthalmic disorders, characterized by structural and proliferative abnormalities of the corneal endothelium, affecting men and women with onset at young to middle age [2]. The etiology of ICE syndrome is not clear, and there is still no effective treatment for halting this disease. A recent hypothesis indicates Herpes Simplex Virus (HSV) infection as responsible for initiating ICE syndrome by the integration of viral genetic material into the human genome [3]. According to this theory, HSV infection is capable of changing the activity and morphology of endothelial cells, allowing them to mitose with abnormal proliferation [3]. Specular microscopy studies [4] and scanning electron microscopy [5] showed that the abnormal endothelial cells of ICE patients acquire an epithelial-like phenotype with the presence of desmosomes, filopodia and microvilli. Immunohistochemistry studies revealed the presence of vimentin and cytokeratin, typical of epithelial cells [6]. This pattern of pathologic characteristics causes progressive migration of the epithelial-like endothelial cells posteriorly beyond the Schwalbe line, obstructing the iridocorneal angle and lining the iris surface, causing progressive iris atrophy, by contraction of a cellular membrane secreted by the abnormal endothelial cells [7].

This pathological condition leads to varying degrees of corneal decompensation, iris atrophy, and secondary angle-closure glaucoma occurring in a high proportion of patients [7]. ICE syndrome comprises a spectrum of three clinical variants: Chandler Syndrome, Essential Iris Atrophy (EIA) and Cogan-Reese Syndrome [2, 8]. ICE syndrome is non-hereditary, and it is usually unilateral affecting adult women more often than men [9].

Few cases of KC associated with ICE syndrome variants have been reported [10–12]. The herein case report describes the clinical features and the complex management of the rare association of KC and unilateral EIA.

## Case presentation

A 38-years-old Caucasian man was referred to the Ophthalmology Unit of the AUSL-IRCCS Hospital of Reggio Emilia, complaining progressive decrease vision in his right eye over the past few years. His history was negative for systemic and ophthalmic diseases, eye rubbing, and allergic conjunctivitis. All the available family members examined were negative for any physical or ocular abnormalities.

On presentation, uncorrected visual acuity was “hand motion” in the right eye, due to a high degree compound myopic astigmatism, and 20/20 unaided in the fellow eye. Following application of a rigid gas permeable contact lens (CL) the visual acuity in the right eye improved to 20/40.

**Fig. 1:**
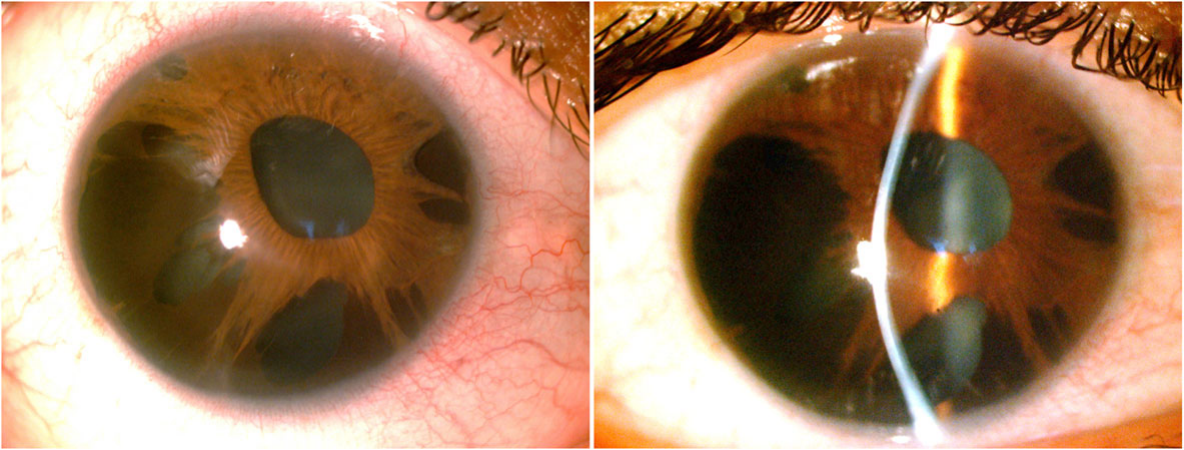
Slit Lamp Examination. Slit-lamp photograph showing clinical features of essential iris atrophy and keratoconus in the right eye

At the slit-lamp examination, the cornea in the right eye showed advanced ectasia with Vogt striae and faint apical scar. Extensive iris atrophy with multiple transillumination defects, mostly in the inferior quadrant was found (Fig. 1). The pupil was oval and decentered super-nasally, with a diameter of approximately 3 mm, poorly reacting to light. Intraocular pressure (IOP) measured by Goldman applanation tonometry was 26 mmHg in the right on topical therapy with timolol 0.5%, brinzolamide 0.1% and travoprost 0.001%. The left eye presented a normal IOP of 13 mmHg.

Gonioscopy revealed the presence of peripheral anterior synechiae for 360° with extensive obstruction of the trabecular meshwork in the right eye and normal open-angle in the fellow eye. Optic disc in the right eye showed severe glaucomatous changes. No fundus alterations were found in the left eye.

Endothelial microscopy (CEM 530 Specular Microscope, Nidek Medical Srl) revealed a severely abnormal endothelium in the right eye with undefined cell morphology. The left eye presented a cell density of 2340 cell/mm^2^ with regular morphology.

**Fig. 2:**
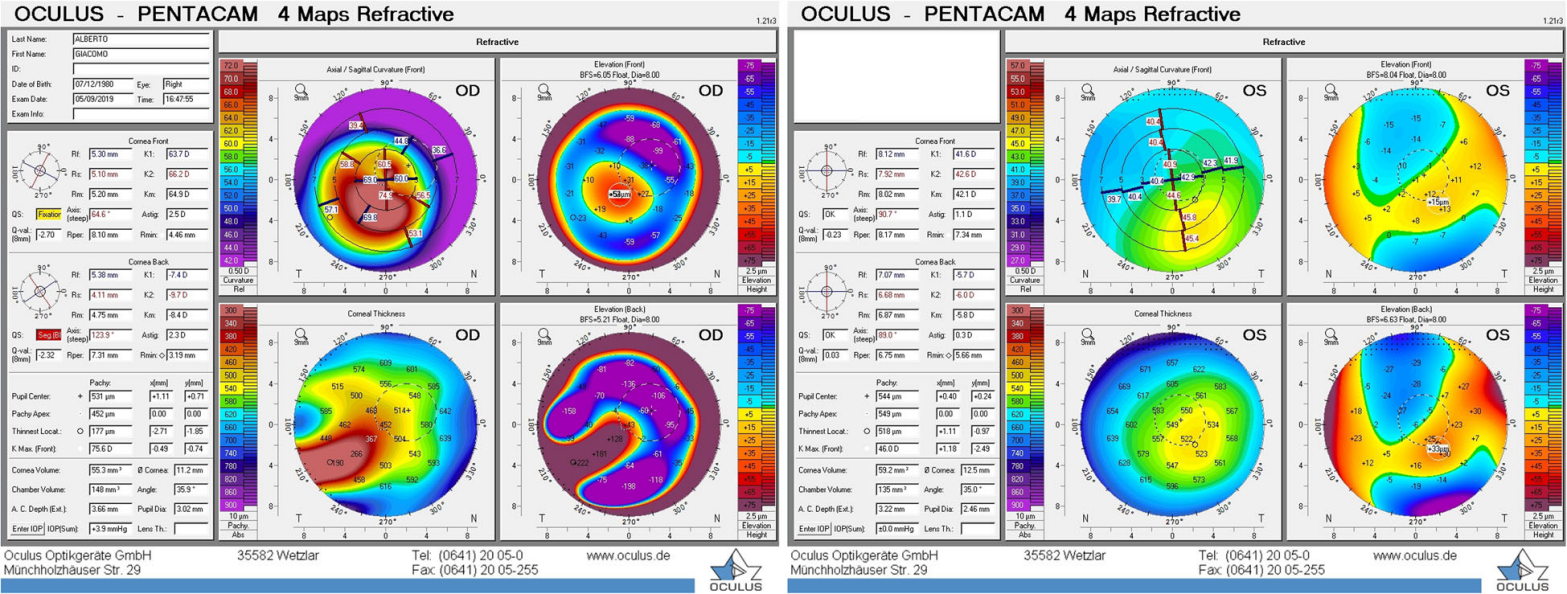
Corneal Tomography. Sheimpflug camera corneal tomography map showing advanced keratoconus in the right eye and forme fruste keratoconus in the left eye

Corneal tomography (Pentacam HR, Oculus) confirmed the diagnosis of bilateral KC with more severe involvement of the right eye. Pachymetry map showed the thinnest point measuring 177 μm in the right eye. The left eye presented topographic signs of a forme fruste KC with a central pachymetry thickness of 549 μm (Fig. 2).

In the right eye, the Humphrey visual field (HFA II 750, Carl Zeiss Meditech Inc., Dublin, CA) showed severe tubular constriction. Visual field in the left eye was normal.

Because of the severe glaucomatous damage in the right eye, the uncontrolled IOP on maximum tolerated medical therapy, and a stable corneal disease a glaucoma drainage device implantation (GDI) was advised.

A 350 mm^2^ Bearveldt tube insert (BGI; Abbott Medical Optics, Abbott Park, IL) was implanted. A fornix based conjunctival flap was lifted, the endplate was positioned in the superior-temporal quadrant and sutured to the sclera at a measured distance of 10 mm from the limbus. The tube was inserted into the anterior chamber away from the corneal endothelium. The limbal portion of the tube was covered with a donor scleral patch [13]. Surgery was uneventful.

GDI implant was positioned well away from limbus not to disturb the CL, that continued to be well tolerated. After 3 months from surgery, IOP reached a stable value of 16 mmHg without anti glaucomatous topical therapy.

## Discussion and conclusions

KC is usually an isolated sporadic condition, although multiple reports described its association with other systemic or ocular disorders. More frequently, KC has been associated with atopic disease [14, 15], eye rubbing [16], floppy eyelids [17], Down syndrome [18], mental retardation [19] and connective tissue disorders [20]. KC has been reported in combination with rare genetic diseases, including corneal dystrophies. Cremona FA et al. published a 10-year review of the concomitant presentation of KC with other corneal dystrophies, but no cases of keratoconus and ICE syndrome were reported [21]. In the literature, only a few cases of KC associated with one of the ICE syndrome variants have been reported [10–12].

The herein case describes the rare association of KC with EIA in the spectrum of ICE syndrome. EIA diagnosis was made based on unilateral involvement, non-familial nature, corneal endothelium morphology and iris abnormalities. All these signs were sufficient to exclude posterior polymorphous dystrophy, which may be associated with KC and should be considered in differential diagnosis with ICE syndrome [22].

Our case poses several clinical challenges regarding KC correction, glaucoma treatment and the risk of corneal endothelium decompensation.

The management of KC, when associated with ICE syndrome, may require peculiar considerations regarding the association with the multiple anterior segment abnormalities present in these patients. Spectacles and, more efficiently, rigid gas permeable CLs should be first attempted for the correction of early and advanced KC patients as they may provide useful to excellent visual acuity [23]. The introduction of corneal crosslinking (CXL) has significantly changed the management of KC [24], allowing to strengthen the corneal stroma in order to arrest or slow KC progression.

In the literature, no cases of CXL in KC patients with associated ICE syndrome were reported, probably because ICE syndrome becomes manifest in the adult age when the KC is unlikely to progress. In the clinical setting, a corneal thickness of 400 μm has traditionally been regarded as the minimum pachymetry to prevent endothelial cells damage after CXL following epithelium removal [25]. In case of KC with ICE syndrome, we consider that this cut off needs to be strictly observed in order to minimize any potential disturbance to the abnormal endothelium. In this patient, CXL was not considered indicated due to the lack of evidence of keratoconus progression, the age of the patient and the low corneal pachymetry.

Keratoplasty is a surgical option for the correction of KC whenever a CL is not tolerated or fail to provide an adequate vision correction. In our case, penetrating keratoplasty (PK) would allow concomitant correction of the corneal ectasia and the endotheliopathy with a single procedure. Full-thickness corneal replacement by PK in ICE patients has been investigated reporting a rate of 83 to 100% with a mean follow-up of 3 years [26–29]. However, graft failure occurred in 83% of the patients with a rejection rate of 30% [30]. Alvim PDTS et al. reviewed the outcome of PK in 14 patients with ICE syndrome with a follow-up of 58 months. Clear grafts were achieved in 12 cases (85%) although six patients (43%) required a second PK [31]. Deep Anterior Lamellar Keratoplasty (DALK) procedure would likely expose the patient to a lower risk of immunologic rejection than PK, by replacing the stroma while sparing the host endothelium. However, no cases of DALK performed in patients with both KC and ICE syndrome are reported in the literature to support this hypothesis and the advantages offered by an endothelial sparing technique in patients with an abnormal endothelium is still questionable.

Endothelial decompensation with corneal edema is the most common cause of decreased vision in patients with ICE syndrome due to the concomitant endothelial abnormality and the raised IOP. The technological evolution of Endothelial Keratoplasty (EK) offers encouraging results for the treatment of ICE syndrome patients with corneal decompensation [32, 33]. Due to the rarity of ICE syndrome and to the lack of comparative studies, it is arduous to state which between full-thickness or lamellar graft is more indicated in these patients. However, in consideration of the several advantages offered by a minimally invasive procedure, allowing for negligible refractive changes, prompt visual recovery with excellent visual outcomes and lower risk of rejection, we support the use of EK in these patients with complex anterior segment disease [34–36]. Sorkin N [37] et al. reported the visual outcomes of a small case series of 4 eyes with ICE syndrome treated with Descemet Membrane Endothelial Keratoplasty (DMEK) with a follow-up of 6 months. They did not report any case of graft failure and graft rejection.

In the herein presented case, an alternative treatment to PK would be a sequential approach using DALK to correct the corneal ectasia and eventually an EK procedure to treat the endothelial decompensation should it develop with time. It is worth to consider that in ICE patients, any keratoplasty technique would not remove the entire abnormal endothelium; therefore, the progression of the baseline disease would not be halted by excising the full cornea thickness. Moreover, it is plausible to consider that multiple keratoplasty procedures may be required during the lifetime of these patients due to the high risk of late graft failure. For this reason, EK more than PK may be the technique of choice because of the easier graft replacement. Although it is well known that DMEK can provide superior visual outcomes than DSAEK, its benefits in patients with severe ocular comorbidities may be questioned. In our opinion, in ICE patients, extensive synechiae, significant iris defects, and the high chances of rapid endothelial cells loss, with the need for repeated EK procedures make DSAEK a more reasonable choice. Currently, our patient has not yet developed endothelial decompensation.

The medical management of glaucoma in ICE-patients is challenging and often represents a temporary solution requiring surgical intervention for optimum IOP control. Topical treatment of ICE syndrome-related glaucoma presents a high failure rate ranging from 60 to 80% [2, 3, 38, 39]. Due to the rarity of this disease, few data are available on the surgical management of glaucoma, coming from retrospective case series. Filtrating surgery in ICE patients showed a lower success rate than in other types of glaucoma ranging from 60% at 1 year to 40% after 2 years [40–42]. This percentage drastically decrease to less than 20% in cases of reintervention [41]. The use of antifibrotic agents is controversial (mitomycin-C, 5-fluorouracil), and it has been attempted to increase the success rate of filtering procedure in ICE syndrome-related glaucoma. Mitomycin C shows to be more effective than 5-fluorouracil in preserving bleb functioning over time [42–44]. Trabeculectomy failure is probably secondary to the ingrowth of endothelial cells in the filtration site responsible for the progressive obstruction [45]. A significant proportion of eyes undergoing trabeculectomy for ICE syndrome (12.5–53.8%) requires a secondary Glaucoma Drainage Implant (GDI) at some stage, suggesting that trabeculectomy does not always offer a definitive treatment [46]. GDI showed a success rate of 70% at 1 year, from 70 to 40% at 3 years, and up to 50% after 5 years [46, 47]. About 20% of eyes were reported to have a proliferation of endothelial cells blocking the tube ostium [47].

Finally, 50% of eyes developed corneal decompensation after 4 to 5 years requiring a corneal graft [48]. Postsurgical inflammation, the use of antimetabolite agent, an altered aqueous humor environment, the turbulence at the tube tip, and tube migration [49] could have an amplified negative effect on the abnormal endothelial cells with the subsequent need of a corneal graft.

In the case herein presented, in consideration of the high IOP, despite maximum medical therapy, the advanced glaucomatous damage and the risk of failure of trabeculectomy failure, a primary Baerveldt implant was performed in order to not jeopardize CLs fitting by the formation of a limbal conjunctival bleb. After a fornix based conjunctival incision, we decided to locate the anterior edge of the valve plate as much posterior to the limbus as possible at 10 mm, in order to preserve the limbal conjunctival tissue. This strategy allowed the patient to continue using CL with no fitting problems and stable visual acuity. A further possibility would have been to place the tube in pars plana. A recent meta-analysis demonstrated that a pars plana vs. anterior chamber tube implantation have a similar incidence of corneal endothelial failure and other complications [50].

The need for cyclodestructive procedures in patients with ICE syndrome-related glaucoma is higher than in other forms of glaucoma since that ICE-patients are young with a higher cicatrizing response causing bleb failure [40].

In conclusion, due to the complexity of ICE syndrome, the management of these patients is challenging. In our case, we chose a step approach by fitting a CL and performing a GDI surgery in order to correct the IOP. Should with time the CL no longer be tolerated, then a DALK would allow correcting the corneal ectasia, whilst preserving the host endothelium until functioning. Eventually, in the case of endothelial failure, an EK may be performed to restore corneal clarity.

## Data Availability

Data sharing is not applicable to this article, as no datasets were generated or analyzed during the current study.

## Abbreviations

CL: Contact lens
CXL: Corneal crosslinking
DALK: Deep Anterior Lamellar Keratoplasty
DMEK: Descemet Membrane Endothelial Keratoplasty
DSAEK: Descemet Stripping Automated Endothelial Keratoplasty
EIA: Essential Iris Atrophy
EK: Endothelial Keratoplasty
GDI: Glaucoma Drainage Implant
HSV: Herpes Simplex Virus
ICE: Iridocorneal Endothelial Syndrome
IOP: Intraocular pressure
KC: Keratoconus
PGs: Prostaglandins
PK: Penetrating keratoplasty
